# Quarterly Summary

**Published:** 1853-10

**Authors:** 


### QUARTERLY SUMMARY.

### DENTAL SCIENCE.

1.—*Treatment of Exposed Dental Pulps preparatory to Filling.*—Professor J. D. White, M. D., in an inaugural thesis on the treatment of exposed dental pulps preparatory to filling, presented to the faculty of the Jefferson Medical College, session 1843-4, and published in the Dental News Letter for July, 1853, after describing the anatomy of the teeth, the structure of dentine, enamel, cementum and the manner of the formation of these several dental constituents, enumerates the membranes of the teeth; then notices the different opinions concerning the organization of these organs; after which he proceeds to show, that when the pulp of a tooth becomes exposed to the action of the fluids of the mouth and atmospheric air, by the loss of a portion of the crown, it soon becomes the seat of active inflammation. When the inflammation is permitted to run its course, the injury sustained by the tooth, the author then goes on to show, is usually so great as to preclude the restoration of the organ, especially if it be a molar, to usefulness. But, as it sometimes happens when the inflammation is mild, and terminates with the sloughing of the pulp at the apex of the root, the tooth may be made to subserve the purposes for which nature intended it, for many years, by being properly filled. Assuming this to be true, he believes it to be the duty of the dentist either to combat the inflammation, or, in all cases where caries has extended to the chamber of the tooth, to destroy the pulp at once with a view of arresting the progress of the disease before the neighboring structures shall have become implicated. This he regards as the least of two evils.

As some authors entertain the belief that where the pulp of a tooth is destroyed, the organ becomes to all intents a foreign body, and must necessarily, if permitted to remain in the mouth, act as a morbid irritant, professor W., enters into an argument to show, that the dental periosteum may supply a sufficient amount of vitality to prevent it from producing this effect. This view of the subject is supported by the fact, that teeth have remained in the mouth for years, with impunity, after the extirpation of the pulp, and the obliteration, by filling, of the chamber of the organ. One example is cited, in which a tooth had been thus preserved for thirty years. The cause of the irritation sometimes observed in the sockets and gums of the teeth, after the destruction of the pulp, the author believes to be principally owing to the admission of air and the fluids of the mouth, through the foramina to the apices of the roots.

As the pulp, after it has become exposed, assumes a pathological condition, the object of the dentist should be to prevent the consequences likely to arise from it, and at the same time to secure the retention of the tooth.

[To be Continued.]

2.—*Filling Teeth over Exposed Nerves.*—Dr. W. G. Oliver states, in the Dental News Letter, July, 1853, that where a nerve is exposed, whether recent or otherwise, he does not hesitate to fill the tooth, provided the cavity will hold a plug. If the tooth has ached, or soreness supervenes, he applies camphorated ether on a bit of cotton, then drills a hole through the organ to the channel of the nerve, carrying the instrument far enough to cut through the nerve; after which, the tooth may be filled in the usual manner. This method of procedure, he says, he has practiced for more than seven years, without having failed in more than one in a hundred cases.

The above method is somewhat similar to that recommended by Drs. Hullihen and Miller.

3.—*Inflammation of Dentine.*—Increased sensibility and excessive tenderness so frequently observed in dentine remote from the pulp of the tooth, professor Townsend, see Dental News Letter, April and July Nos., 1853, regards as certain indications of inflammation. The reason that all the phenomena characteristic of a pathological condition of this sort in other parts of the body, such as increased heat, redness and pain, is not observed here, is owing to the fact that the vessels of dentine are too small to circulate red blood, and the unyielding nature of tooth-bone will not admit of their expansion. Sound teeth are susceptible to impression both from sweet and sour fluids, while a tooth deprived of vitality is not endowed with such attributes, a dead tooth is generally more susceptible to impressions from heat, a fact which would seem to favor the idea that it was a better conductor, and that it has lost the power of regulating its own temperature, a power belonging to healthy living parts. The truth of this is shown by a case recorded by Dr. George McClelland, who in the extirpation of the parotid gland, removed the portio dura of the seventh pair of nerves, causing paralysis of the cheek. Afterwards, the cheek was so remarkably susceptible to impressions of heat and cold, that in a hot room, it often became necessary to apply cold water, while the sound cheek was not unpleasantly affected. When exposed to a low temperature, the paralyzed cheek had to be defended from the cold.

Inflammation of dentine may be destructive, and produce caries or ulceration, but when the diseased part is removed, the surrounding sound bone will not evince any sensibility. The most sensitive portions of a tooth are often near the surface or immediately beneath the enamel, at the extremities of the nervous fibres, where the largest amount of sensation appears to reside, the larger branches serving only as so many conduits for the conveyance of the vital fluid, and to convey impressions from the extremities to the brain. Lesions of the crown of a tooth, whether the result of mechanical violence or the action of corrosive agents increase the susceptibility of dentine to painful impressions.

"But when the dental nerve and artery are removed by the excavation and clearing of the fangs, something is taken away that is, more or less, essential to the life of the tooth? Certainly. It is not pretended that parts having such offices as those which make up what we call the pulp, are merely indifferent to the constitution and functions of the tooth. If the common motor and sensitive nerve of a muscle be cut, compressed, or removed, the sensibility to external impressions, and the power of voluntary motion, which they previously conferred upon the muscle, are lost. The muscle is palsied. Is it, therefore, dead? No. It lives as an individual organism, but it has lost its instrumental uses. These uses were its mobility and that sensibility to external impressions which are conveyed to the brain or seat of consciousness. But the mobility proper to its arteries, veins and lymphatics is untouched, and its organic sensibility remains unhurt, for every fibre retains the appetencies, or organic sensibility which is concerned in nutrition, growth and the reparation of parts. It, the muscle, lives on, as the stomach, liver, bowels, lacteals and great arteries in the abdomen and thorax live.

"The processes essential to its own peculiar vitality are carried on as before. All that has happened is the loss of voluntary motion and consciousness, and the consequences of these, whatever they are: it is simply a paralyzed member, paralyzed in motion and perception—these are essential to its related functions. But how is it with a tooth? It has no voluntary motion proper, and the loss of a nerve of the motor kind, leaves it capable of playing anvil, cutter, grinder or crusher as before. Its normal character is immobility and solidity. These properties conform it to its active functions or animal uses. Most probably the maxillary branches of the fifth pair of cerebral nerves, are not motor nerves at all: one branch of this same pair is regarded as a nerve of taste, or gustatory nerve, while it is the ninth pair which give voluntary motion to the tongue. I think it probable enough that the lingual branch of the fifth pair has the function of taste, because the two other branches of this great trigeminal trunk are distributed to the teeth, most probably to endow them with that kind of sense which is nearly allied to taste. If so, the harmony of their office with that of the tongue, in alimentation, would be explained, as well as their perception of sweet and sour, which we certainly have by the teeth, though it is the tongue which enables us to give them their fully distinct recognition. It is, I

believe, a well established doctrine, that nerves coming from the same tract or cerebral or spinal mass, have like functions. If one branch of the fifth pair is the gustatory nerve, the functions of the other two, the upper and lower maxillary branches, very likely, have some office analogous to this. That is its sensitive root, for it has a sensitive as well as a motor root, like the spinal nerves, (and it is the only one of the cerebral nerves that has such double root.) Its sensitive root may have the function of taste, and the motor division be designed to supply voluntary motion to the skin and muscles of the eyelids, nose, lips and jaws, to which branches are distributed; indeed, its distribution upon the face and bones, and its connection with the organs of the senses, is so general, that it has been called the great sympathetic nerve of the face. Certain it is, that the teeth get no voluntary motion in their sockets from the maxillary nerve, and it is much more probable that the sensitiveness which it gives, is rather related to taste than to the perception of the common properties of bodies, such as the fingers and skin possess. The sense of resistance, or the perception of hardness and softness, may be had in the gums, or by the pressure of the cheeks, tongue, palate, lips and other parts; and the modification of touch which we have in the mouth, for the purposes of mastication, can be well supplied by parts situated there, other than the teeth; indeed, their great solidity makes them unfit to be impressed by the mere tact of bodies, while the soft parts of the mouth may better serve that purpose.

"Now, putting all these speculations together, and adopting them, for want of better light upon the subject, we will have the tooth whose pulp is destroyed, deprived only of its modified sense of taste; for touch, in the strict meaning of the word, and motion, it has not; and this deprivation will leave it adequate to all its uses as a masticating organ, and the use it serves as a part of the vocal apparatus; and, if only its organic life can be maintained, it may be allowed to remain, with excellent right to the room it occupies. So far, we have it a grinder or cutter, and a reverberator or warder of vocal sound, as good as ever, and only deprived of its sense of feeling, whatever that be, whether dental taste or whatever else it may be termed. Its continued existence, with this defect, (which defect the remaining teeth may very well supply,) will depend upon the maintenance of the circulation in it, of sufficient perfectness for the purposes of nutrition, and the absorption which goes hand in hand with the sanguineous supply in all living parts. To secure this, it is only necessary that the blood-vessels which reach the tooth externally, through the medium of the investing membrane or periosteum, be alive and healthy; for, the subsidiary inosculation between the capillary branches of the internal and external arteries, will keep all the interior abundantly supplied, under a provision of nature which meets the changes of action which occur continually, and those interruptions and destruction of vessels which happen as incidents occasionally. The capillaries which remain connected with their trunk enlarge, and assume the double duty of supplying both sides or sections of any part that needs such accommodation. Thus, then, that interior portion of the tooth which the dental artery at first furnished with the nutritious fluid, may be sufficiently supplied, and thus, I have attempted to give some of the reasons for the possibility of a tooth remaining a useful member of the mouth, after it has been deprived of its primitive internal circulation."

---

4.—*Extraction of Teeth.*—The continuation of Dr. J. Taylor's article on the extraction of teeth, as published in the Dental Register, April No., 1853, we copied entire into the last (July) No., of the Journal, and if space permitted, we would transfer the entire paper to our pages, but as we cannot do this, we present the following summary of the remainder, contained in the July No. of the Register.

The forceps enumerated by Dr. T., for the extraction of the teeth of the upper jaw, as described in the article copied into the last No. of the Journal, consist of four molar forceps, two for either side, a dens sapientiæ, and a bicuspid forcep. To these he adds a small pair for the lateral incisors, and two pair for the molars—one for either side, with the outer beak brought to a point to pass between the buccal roots.* For the lower molars, he recommends the use of two pair—one for the right and one for the left side. The right is a hawk's-bill, one beak passing over the crown of the tooth is adapted to the lingual surface. The bar of the instrument is slightly curved, to enable the operator to grasp a second or third as readily as a first molar. The end of one of the handles is curved, passing round the little finger. When the instrument is in the hand of the operator, the beaks are fitted to the necks of the teeth, with a central point to each to pass between the roots of the teeth, so that when these are straight, the mere closing of the instrument will sometimes raise the organ from the socket. The beaks of the instrument for the left side, are adapted in the same manner, but is put together differently. It opens to the right and left instead of up and down, and has two curves, the beak being turned downwards. It is also bent near the joint, or rather between this and where the handles are fitted to the hands, in a direction towards the operator.

* These forceps are known as Maynard's forceps.

The hawk's-bill forcep, (with the ends of the beaks crescent, we presume,) is used for the bicuspids and cuspidatus of the right side, and straight or curved root forceps for the incisors.

For the removal of the lower dentes sapientiæ, he recommends what is usually called Physic's Elevator*—an instrument shaped something like a pair of forceps. The blades vary in length from half an inch to an inch. They are concave on their outer and convex on their inner surface, and bent to near a right angle with the handles of the instrument. The blades are applied between the second molar and wisdom tooth, lifting the latter, as they are forced together by closing the handles, from the socket. The blades, however, should be forced firmly against the wisdom tooth.

But the use of this instrument is only applicable when the approximal surfaces of the second molar and wisdom tooth are sufficiently sound to resist the pressure. Nor can it be employed when the second molar is wanting, or has become very much loosened in its socket. After the dens sapientiæ has been raised from its socket, it is usually necessary to have recourse to a pair of forceps for its removal.

The forceps employed by Dr. T. for the extraction of roots of teeth, consist of three or four pair, with very sharp, well-tempered beaks, varying in their curve from straight to nearly a right angle.

For the extraction of a lower molar on the right side, the author adopts the following method of procedure: he stands a little to the right and behind his patient, on a stool, then adjusts the instrument to the tooth, and with an upward and outward movement—an inward motion, with this description of instrument, to any extent, being inadmissible when the crown of the tooth stands inward, the tooth is loosened and lifted from the socket. But when the roots are much separated, as is often the case with the first molar, it may be necessary to move it inwards, outwards and backwards, before it can be extricated. When the crown is very much decayed the instrument should be forced upon the neck as far as possible, to prevent the liabily of breaking it, applying, at the same time, an outward and inward motion, before exerting any direct extractive force. If, notwithstanding, the crown breaks below the edge of the gum, the roots are removed with one or other of the forceps

*Dr. Physic was indebted for the idea of this instrument to Dr. L. S. Parmly, who exhibited it to him soon after he invented it. It should, therefore, be called Parmly's Elevator.—*Eds.*

already described for their removal, securing a firm hold by forcing the beaks down to the edge of the alveolus. If the crown breaks down to the edge of the alveolus, the gum is separated and the socket cut away sufficiently to admit of the application of hawk's-bill forceps, with a single pointed beak, securing a hold between the roots, then, with an inward and outward motion, they are loosened and raised from the socket. If one should remain, it may afterwards be easily removed with a pair of root forceps. It sometimes happens that the disposition of the roots is such as to render their separation from the crown, previously to removal, absolutely necessary. An example of the kind is given by our author.

5.—*Irregularity of the Teeth.*—In a paper read before the Mississippi Valley Association of Dental Surgeons, published in the July (1853,) No. of the Dental Register, Dr. Hamell expresses the belief, that irregularity of the teeth is the result of functional derangement during dentition, and that its existence increases the liability of these organs to disease, and is productive of an unhealthy action in the parts with which they are immediately surrounded. In view of these considerations, the author urges the importance of more attention, on the part of dentists, to orthodontia, than is usually bestowed upon this specialty of dentistry.

The period when the resources of art can be most efficiently applied in remedying deformities of this kind, is, he says, previously to the sixteenth year, although it may often be had recourse to, successfully, at a much later period. The means must be varied to suit the peculiarities of each individual case.

6.—*Caries of the Teeth.*—In the April number of the Journal we gave a brief summary of the views of Professor J. Taylor, as expressed in the first part of his answer to certain interrogatories proposed to the late Dr. Drake, on the subject of caries of the teeth. The April and July numbers of the Dental Register contain the remainder of Prof. T's reply.

The premature decay of the temporary teeth, he attributes to physical imperfection, derived from the parent, and to chemicalaction of vitiated secretions of the mouth, arising from constitutional disease, or other corrosive agents. The truth of this opinion has been abundantly confirmed by observation, and that the greater liability of the deciduous, than the permanent teeth to decay, is attributable to the less perfect organization of the former than the latter, and to their more constant exposure to the action of diseased buccal fluids, and other external destructive agents, such as acidulated candies, &c.

In reply to the second question propounded by Dr. Drake, "To what causes, external or pathological, local or constitutional, shall we ascribe the premature decay of the second teeth in the west," and, "Is a hereditary, scrofulous diathesis a cause of infirm teeth?"

To this question, Dr. T. replies, in substance, that the operation of a constitutional cause, which would affect, injuriously, the physical condition of the temporary teeth, would operate similarly upon the replacing organs. He notices the influence of diet and constitutional disease on the functions of the formative organs of the teeth. He next alludes to the effects of a disordered condition of the alimentary canal upon the secretions, and, as a consequence, the irritable condition of the gums, which, around the necks of the teeth inflamed, the mucous secretions, under such circumstances, are acidulated, and when left on the teeth, act upon the parts with which they are in contact, causing decomposition of their earthy salts. This effect is the more marked on those parts where it remains longest, and is not freely mixed with saliva, and the dentine and enamel are not very close and compact in their texture. Improvement of the state of the constitutional health is followed by a lessened tendency of the teeth to decay. Thus, an "hereditary, scrofulous diathesis" is regarded as "a cause of infirm teeth."

Among the external local causes not yet enumerated, are the oxalic, sulphuric and succinic acids, all of which have a stronger affinity for the lime of the teeth than the phosphoric, with which it is combined. The citric also acts upon it, though not as rapidly as do nearly all the other acids, both vegetable and mineral.

A crowded arrangement of the teeth, favoring the retention of corrosive agents, contributes to the decay of these organs.

In answer to the question, "Is dyspepsia a cause of early decay?" and, "does the acid thrown up by many dyspeptics, in paroxysm of that disease, act chemically on the teeth?" Dr. T. notices, first, the acid condition of the secretions of the mouth in persons afflicted with this disease, then enters into an investigation of the chemical qualities of the gastric juice, both in health and disease, referring to the researches of Schwann, Prout, Gmelin, Tiedeman, Simon, Hünefeld, Dungleson, Blondlot, and others, which, though the results are somewhat varied, all tend to establish the existence of a free acid. From the facts thus established, he arrives at the conclusion, that when the gastric fluid is brought in frequent contact with the teeth, its effects are injurious to them.

7.—*Springing of Plates.*—Dr. S. D. Muse recommends, in Dental Register, July No., 1853, the enclosure of a piece about to be soldered in an iron wire, about a line in diameter, bent in the following form, ΛΛΛΛ. Thus prepared, a piece of sufficient length to form a belt to enclose the piece, is put in shape by bringing the two ends together and fastening. The width of this belt should be about as thick as the iron band usually employed. This belt is now encircled by another piece of wire of the same size, about one-fourth of an inch all around; it is next made fast at opposite points with a piece of wrapping wire, using the precaution to draw it as tight at one point as at another. This done, a rim of pasteboard is placed around the belt, a paste of sand and plaster is poured in until the piece is imbedded. This having set, the pasteboard rim is removed, and the ties clipt which bind the outside wire to the belt. When the paste has thoroughly dried, the work is placed on the apex of a charcoal cone built over a fire on the hearth, solder and borax having been previously applied. The piece heats up evenly and gradually. During this process, burning coals are placed around it, keeping one the whole time over the teeth. As soon as the mass is at a lively red heat, it is placed on a piece of charcoal, scooped out and well ignited, when in a short time the soldering is completed, and the plate not sprung.

The advantages of the wire-belt over the iron band, is, that it does not crush the mass or derange the work in contracting, and it yields to the expansion of the plate, and is less cumbersome.

8.—*Plate Teeth.*—Dr. J. S. Clark, dentist of New Orleans, says in Dental Register, July, 1853, he never secures a plate in the mouth by means of clasps passed between remaining natural teeth, but depends on a mere bearing against the palatine surface on each side, and perfect accuracy of adaptation for the retention of the piece, and carrying it back as far as possible for the bearings, that the average of the base may bear a large proportion to the average of the tooth. In some cases, the bearings are made wholly on fillings introduced expressly for the purpose. The object of this is to prevent injury to the natural teeth resulting from the retention of the secretions of the mouth between them and the clasps.

In applying backings to teeth, he objects to covering the whole of the palatine surface, as it gives to them an opaque appearance. In replacing the loss of bicuspids, artificial cuspids are in many cases preferable to substitutes with two cusps.

9.—*Application of Teeth on a Plate to the Roots of Natural Teeth.*—Dr. J. S. Clark, recommends in Dental Register, July 1853, the application of plates with artificial teeth to roots of the teeth to be replaced. They are first, thoroughly cleansed, filled and filed down to the gums. This method, he believes, to be preferable to the removal of the roots, as the natural appearance and contour of the mouth will be more effectually preserved, and without any injurious effects resulting from their retention.

10.—*Intermittent Odontalgia.*—Dr. H. R. Smith, dentist, of Terre Haute, Ind., relates in the Dental Register, for July, 1853, the case of Mr. G———, aged forty, who suffered severe paroxysms of pain in a right superior bicuspid, which came on regularly at 8, A. M. and at 7, P. M. every day, each lasting about two hours. He was completely relieved by a cathartic of calomel, and the free use of quinine.

11.—*Blow-Pipe.*—J. B. Williams, dentist, of Monongahela City, Pa., describes in the Dental Register, July, 1853, a permanent blow-pipe, capable of producing a constant, steady blast, of any desired strength.

"It consists of three short zinc cylinders, placed one within another, thirty-six inches in length. The largest cylinder is twelve inches in diameter; the inner, smallest cylinder, is eleven-and-a-half inches in diameter; these are soldered together at the lower end, leaving a narrow space of about one-fourth or one-half an inch between them. This space is partly filled with water, and the third or middle cylinder being closed at the upper end, is made to pass down into it, and nearly adjusted to the inner surface of the outer cylinder, so as to pass between it and the inner one without much friction."

"The smallest inner cylinder is closed also at the upper end, except a small opening at the centre, in which a one-half inch lead pipe is soldered, that passes out at the open end below, and taken up through the end of the work bench, where a brass stopcock to regulate the blast, is screwed into the end of the pipe."

To this a gum elastic tube eighteen inches in length, with a jet pipe at its outer end, is fastened, and kept in any desired position by means of wires.

"A strong cord passed over a six inch pully, fixed to the ceiling, is attached to a head of wood one-and-a-half inches thick, placed within the upper end of the movable cylinder, the other two cylinders being stationary, raised about twelve inches from the floor; upon this head weights are placed. About one-fourth inch of the outer edge of the movable middle cylinder, is bent in upon the head of the wood to hold it firmly."

A valve, accurately fitted by being turned in a lathe, three-fourths of an inch in diameter, one-half inch thick, with a stem five-eighths of an inch in diameter, three inches long, and made of zinc, is passed through the head of wood, soldered to that of the zinc within, the valve being inwards. A coil of wire is placed around the stem, attached at its upper end, of sufficient strength to hold it up gently. Thus arranged, the whole is worked with one hand, while the other is at liberty. A fluid lamp, having a flat tube two inches long, three-fourths of an inch broad, and one-fourth thick, beveled at the end, with a close fitting slide and cap, projecting from its side near the top, is used.

A small spigot is placed at the bottom of the stationary cylinder, by which the water may be drawn off in very cold weather, to prevent it from freezing.

Dr. W. states that he found it necessary to place a cylinder of wood within the inner cylinder, as the power of the blast is so great, to prevent it from collapsing.

The apparatus, he thinks, may be made at a cost of about ten dollars.

12.—*Risodontryphy.*—F. Y. Clark, dentist, of Griffin, Georgia, says in Dental News Letter, July No., 1853, that he has performed the above operation thirty times, and has only failed in three cases. In performing the operation, he uses nothing but a simple finger drill, made from a common excavator, making the point spear-shape, as recommended by Dr. Hullihen, with one cutting edge a little longer than the other. The advantage of an instrument of this kind over a bow-drill, is, that it leaves one hand at liberty to control the head, and the motion and pressure can be more readily lessened as the point of the instrument approaches the nerve cavity, whereby, small particles of dentine may be prevented from entering the cavity with the point of the instrument.

13.—*New Odontalgic Remedy.*—Dr. D. R. Whipple recommends, in July No., Dental News Letter, 1853, the oil of turpentine, (oleum serebinthinæ) as a remedy for tooth-ache, applied on a pellet to the affected tooth, securing it with a little dry cotton. It is particularly applicable in cases where the pain results from inflammation of the nerve, and from irritation produced by wounding the pulp. In such cases, it affords almost immediate relief.

14.—*Swaging Plates.*—D. H. Taylor, dentist, of Newark, Ohio, adopts the following method for making castings and swaging plates: An impression having been taken, the first metallic cast is obtained in the usual way, it is coated with a thin batter, made by mixing whiting in a thin solution of gum arabic in water, to the thickness of the plate to be swaged. When this becomes dry and hard, tin or zinc is melted and poured on the model thus prepared. When cold the pieces are separated, and the whiting washed from the first casting. Between these, the plate is accurately swaged, and when gotten up in this manner, it is not liable to spring. *Dental News Letter.*

15.—*Preparing the Mouth for full sets of Artificial Teeth.*—In the Dental News Letter, July, 1853, A. T. Williard, dentist, of Chelsea, Mass., recommends clipping off the apices of the gums between the sockets of the teeth, then dissecting back the fibro-mucous tissue from the alveolar border, and cutting off from a fourth to three-eighths of an inch, so as to admit of the edges of the mucous tissue being brought together. The advantages of this method of procedure are stated to be, first, it diminishes the amount of inflammation; second, it saves nature the task of removing the parts thus taken away; third, it destroys the mucous follicles along the alveolar border, thereby expediting the consolidation of the parts; and lastly, the gums will be in a proper condition for the reception of artificial teeth, four or five months sooner than they would, were the removal of the edge of the alveolar ridge left to the operation of nature, and besides, it has a better shape for the reception of a dental substitute.

16.—*The Teeth, in respect to Character.*—In an article under this head, published in the August (1853) No., of the New York Dental Recorder, Dr. A. Hill, the junior editor and author, after a few interesting and very appropriate introductory remarks, states, that if we were permitted fully to comprehend the entire relationship existing between the different organs of the human system, one class of organs would reveal to us the peculiar character of each; and thus, by witnessing *one* fact, we could easily infer many important considerations pertaining to it. But since our knowledge is necessarily so limited and imperfect, and the action of one class of organs so modified by another, we can only hope to approximate the truth somewhat remotely.

Referring to the fact, that CUVIER, the great naturalist, from his knowledge of the relationship of the different parts of the body, was enabled to reconstruct the animal of an extinct species from a mere fragmentary part of the frame work, and to determine the peculiar habits of the animal, its mode of life, food, &c., he believes, that much may be inferred of the character of an individual from the shape, size, position, and number of his teeth, and that these determine the shape of the dental arch, to which the lips correspond. These features must ever hold a controlling influence over the countenance, and the characteristics of the mouth and teeth constitute the strongest peculiarity by which the inferior animals are distinguished.

It is frequently instinctively said, on beholding an individual for the first time, that person has a very benevolent expression, that fellow looks miserably mean. The different and conflicting traits of character, are distinctly and instinctively read. The flat nose, thick lips, elliptical arch and large herbiverous teeth, are distinctive of the Africans, and to these correlative signs, the dental organs are specially related, and are altogether highly expressive of character. If these signs were removed, and their relations dissolved, the African would cease to be African.

Mr. Levison says: "If a number of national crania, were placed promiscuously on a table, I would undertake to arrange them ethnologically, according to their comparative degrees of civilization, merely by the form of their jaws, and the position of their teeth." If such marked changes are produced on the dental organism, with the resulting change in the nose and lips, a corresponding change of character may reasonably be inferred. These controlling signs are not more expressive in the negro, than in the North American Indian, or the Indians of the South Pacific Ocean. The distinction is not more marked between the teeth of the herbivorous and carnivorous animals, than between the teeth of the genuine African, and the native Feegean, and with the distinctive signs of which, there is a perfect correspondence and agreement of character. Thus, the ethnological signs furnished by the dental organism alone, are sufficient to mark the different degrees of civilization in the individual in which they are seen, as well as his peculiar distinctive traits of character. The preponderance of any special trait, may sometimes be so modified by circumstances as to render the signs somewhat difficult of recognition.

The peasantry from some of the rural districts of Ireland, especially among the females, furnish some of the finest specimens of dentition, both with respect to maxillary expansion and the development of the teeth—a crowded or irregular denture being comparatively rare among them. The physiognomical indications of a broad palatine arch, teeth regularly arranged, a finely expanded mouth and voluptuous lips, are expressive of noble and generous character, and constitute symbols that can never be associated with *essential meanness* or *sordid selfishness*. From such a mouth, language comes with the greatest fluency, and is invested with a charm which no other circumstance can impart.

"The broad grin, the mouth stretching from ear to ear, as it were, and the teeth circling in well developed order in the form of an ellipse—the cheeks dimpled in one place and puffed out in another, just ready to explode with real good nature, all conspire to represent the very soul of cheerfulness and benevolence.

"But where lines are drawn in an opposite direction, they most certainly represent an opposite character. And apropos, of a contracted mouth and puckered lips, are the tight drawn strings, and gathered border of a close drawn purse."

### PHYSIOLOGICAL CHEMISTRY.

17.—*Saliva.*—In a review by Dr. Day of Bidder and Schmidt's recent investigations, we find an admirable account of the latest additions to our knowledge of the digestive fluids. The article on the subject of saliva, published in our last number, renders it unnecessary for us to do more than give those results which have been attained since its publication.

We find in this review, an analysis of saliva, by Jacubowitsch, which we subjoin:

| | | |
|---|---|---|
| Water, | | 995.16 |
| Solid constituents: | | |
| Epithelium, | 1.82 | |
| Soluble organic matter, | 1.34 | |
| Sulphocyanide of potassium, | 0.06 | |
| Fixed salts, | 1.82 | |
| | | 4.34 |
| | | 1000.00 |

The 1.82 of fixed salts, consisted of phosphate of soda 0.94, lime 0.03, magnesia 0.01 and chlorides of sodium and potassium 0.84.

The "soluble organic matter" has been usually called *ptyalin,* and supposed to be the most energetic of the saccharifying constituents of the digestive fluids. Bidder and Schmidt do not believe it to be identical with the ptyalin of authors, or the salivary diastase of Mialhe, which they consider to be the product of the admixture of other substances.

The *sulphocyanide of potassium* was found by Kletizinsky to be most abundant after meals, and most deficient towards night. It is diminished by fasting, alcoholic drinks, iodine, salivation, and chronic disease. It is increased by spices and condiments, Peruvian balsam, musk and mental or emotional excitement. In infancy, old age and the later months of pregnancy, it is deficient.

Jacubowitsch has published analyses of the saliva of dogs, which we subjoin for the purpose of comparison.

*a.*—Ordinary or mixed saliva.

| | | |
|---|---|---|
| Water, | | 989.63 |
| Solid residue, | | |
| Epithelium and soluble organic matter, | 3.58 | |
| Phosphate of soda, | 0.82 | |
| Chlorides of sodium and potassium and sulphocyanide of potassium, | 5.82 | |
| Phosphates of lime and magnesia, | 0.15 | |
| | | 10.37 |
| | | 1000.00 |

*b.*—Saliva, excluding the parotid secretion.

| | | |
|---|---|---|
| Water, | | 990.48 |
| Solid matters, | | |
| Epithelium and soluble organic matter, | 4.25 | |
| Phosphate of soda and chlorides of sodium and potassium, | 4.08 | |
| Phosphates of lime and magnesia, | 1.19 | |
| | | 9.52 |
| | | 1000.00 |

*c.*—Saliva, excluding submaxillary secretion.

| | | |
|---|---|---|
| Water, | | 988.1 |
| Solid residuum, | | |
| Epithelium, | 2.24 | |
| Soluble organic matter, | 5.04 | |
| Phosphate of soda and chlorides of sodium and potassium, | 4.20 | |
| Phosphates of lime and magnesia, | 0.42 | |
| | | 11.9 |
| | | 1000.00 |

*d.*—Saliva, excluding parotid and submaxillary secretions.

| | | |
|---|---|---|
| Water, | | 991.45 |
| Solid matters, | | |
| Epithelium and soluble organic matter, | 2.89 | |
| Phosphate of soda and chlorides of sodium and potassium, | 4.50 | |
| Phosphates of lime and magnesia and carbonate of lime, | 1.16 | |
| | | 8.55 |
| | | 1000.00 |

*e.*—Parotid saliva.

| | | |
|---|---|---|
| Water, | | 995.3 |
| Solid residue, | | |
| Epithelium and soluble organic matter, | 1.4 | |
| Phosphates of soda, chlorides of sodium and potassium and sulphocyanide of potassium, | 2.1 | |
| Carbonate of lime, | 1.2 | |
| | — | 4.7 |
| | | 1000.00 |

These fluids contain no albumen, casein, chondrin nor pyin.

The *buccal mucus* contains, according to Jacubowitsch, who obtained it free from saliva by tying all the ducts before he experimented:

| | | |
|---|---|---|
| Water, | | 990.02 |
| Solid residue, | | |
| Organic matters, soluble in alcohol, | 1.67 | |
| " " insoluble " | 2.18 | |
| Fixed salts, | 6.13 | |
| | — | 9.98 |
| | | 1000.00 |

This fluid was viscid and frothy, extremely turbid (from the presence of a number of epithelium cells which did not settle, on standing,) and strongly *alkaline* in its reaction.

In regard to the *quantity* of saliva secreted in a given time, this memoir of Bidder and Schmidt has added much to our knowledge. These observers found that a dog weighing 16 kilogrammes (about 35 pounds) furnished in one hour from one of Wharton's ducts, 5.64 grammes* of saliva, so that the two submaxillary glands yielded in that time 11.28 grammes. One of the ducts of Steno discharged, in an equal time, 8.79 grammes of a clear, limpid secretion; the two parotids, therefore, secreted 17.58 grammes. Assuming that a man weighs four times as much as the dog, or 140 pounds, and that these secretions vary directly with the weight, the hourly secretion of the submaxillary glands would be 45 grammes, of the parotids 70 grammes. The daily secretion of both sets of glands, therefore,

* A gramme is nearly 15½ grains—or more exactly—15.434 grains. These weights, first adopted by the French, are retained because of their almost universal use in chemistry.

would be about six pounds, or a little more. Bidder and Schmidt think that a man actually discharges about half that quantity. They found, by direct experiment, that a man discharged from 100 to 120 grammes an hour. Three pounds may, therefore, be assumed as the average daily secretion.

The *physiological function* of the saliva may be regarded now as fully determined. The doubt which still overhung that question appears to be cleared up and all its difficulties disposed of. The conversion of the *amylacea* into dextrine, sugar and lactic acid, and the facilitation of the absorption of this class of food is clearly the use of this fluid, stationed at the inlet of the body.

The time required for this change is important to be known in estimating the physiological importance of the saliva. If a fresh decoction of starch, proved to be free from sugar, be mixed with an equal quantity of fresh saliva and the mixture agitated, it will instantly become thin and watery. On testing it, iodine no longer gives a blue tint, and Trommer's test clearly establishes the presence of sugar.

"The almost instantaneous induction of this action is a point which must not be overlooked in considering the question whether this is a special property of the saliva, or whether it is shared by other animal fluids. There can be no doubt, as we shall presently show, that in this respect the pancreatic and intestinal juices exactly coincide with the saliva, but when we find stress laid upon the circumstance that many other organic substances, as, for instance, nasal mucus, pieces of kidney, putrifying serum, &c., produce similar changes in eight or twelve hours, at 100° or upwards, we must recollect that at such a temperature, and after so long an interval, changes may be spontaneously set up in a solution of starch. There are, however, a number of animal substances which occasion the appearance of sugar, in a solution of starch, in so short a space of time, as altogether to exclude, in such cases, the suspicion of spontaneous metamorphosis; but the action induced by the saliva is incomparably more rapid even than that of any of these substances."

Bidder and Schmidt performed an extensive series of experiments to compare these other organic matters with saliva. They found that the saliva of adult men and their nasal mucus,* the saliva of a

* It must be observed that a little saliva might be mixed with nasal mucus, in sneezing, &c.—and that in other experiments with nasal mucus the change did not commence till after a quarter of an hour, or more, had elapsed.

child, aged four months, the saliva of dogs, their pancreatic juice and tissue, the parotid and pancreatic tissue of an adult pig, and the gastric juice of dogs which had been rendered alkaline by their swallowing the saliva, all induced the immediate formation of sugar in a solution of starch; but it was only in adult human saliva that the whole of the starch was so changed that iodine produced no blue tint with the mixture. With the other substances, the complete change was effected in various periods, the longest time being one hour. The saliva of a dog, excluding the parotid secretion, brought about this change in twenty minutes; mucus from the urinary bladder of a pig, in thirty minutes; pancreatic tissue of a dog ten days old, in forty minutes; tissue of the submaxillary gland of an adult pig, in an hour; hepatic tissue of the same animal, in an hour and twenty minutes; muscular coat of the bladder of the same animal in an hour and a half; tissue of the submaxillary gland of a dog ten days old, two hours and fifteen minutes; parotid tissue of the same animal, in three hours; acid gastric juice of dogs in which there were no buccal epithelium cells, in one hour and thirty minutes. Mucus from the mouth of a dog whose salivary ducts had been tied a fortnight before, aqueous extract of his detached buccal mucous membrane, his parotid and submaxillary tissue, gave traces of sugar after three or four hours, but the solution of starch remained thick and viscid. Traces of sugar were found eight hours after the parotid and submaxillary secretions were separately mixed with solution of starch. When the secretion of a dog's orbital gland was used, no trace of sugar was found after seven hours had elapsed; when saliva, from which the submaxillary secretion was excluded, was employed, no sugar was formed after two hours, and fifteen hours were sufficient to induce this change in starch subjected to the action of the acid gastric juice of a dog whose parotid and submaxillary ducts had both been tied.

The result of these inquiries, is, that *no one* of the secretions of the mouth, by itself, furnishes the peculiar ferment, but, that this has its source only in the admixture of the secretions.

To determine the relative importance of these different liquids, Jacubowitsch instituted several experiments. He tied the salivary ducts, and found, contrary to Bernard's statement, that the mucous secretion of the mouth could not change starch into sugar. When, however, the ducts of but a single pair of glands were tied, and starch digested in the secretion then obtained from the mouth, sugar made its appearance in five minutes. The parotid and sub-maxillary secretions, however, when mixed, were deficient in this power.

Bidder and Schmidt found that parotid saliva, mixed with buccal mucus, exerts no marked action on the conversion of starch into sugar, while the sub-maxillary secretion mixed in the same manner, acted as promptly as common saliva. They agree with Bernard, in regarding the parotids as furnishing a secretion, simply designed to moisten the dry food, and the submaxillary forms with the buccal mucus the peculiar ferment of the saliva.

Another important result obtained by these observers, is the fact of the inactivity of these glands during infancy. Several experiments determined this point. 1. On establishing fistulous openings in Steno's ducts in calves, no fluid escaped through the canula. 2. Starch was converted into sugar in the presence of the tissue of the parotid glands of adults, in a far shorter time than when the same tissue of an infant's gland was used. 3. When the saliva of an adult man, and that of a child at the breast, were respectively mixed with equal parts of a thick solution of starch, the metamorphic action *commenced* in equally short spaces of time, but it was not *completed* in the child's saliva till a full hour had elapsed, whereas, in the man's, the action was over almost as soon as it had begun.

The necessity of alkalinity to the performances of the functions of the saliva is also considered, and it is ascertained, that this reaction is not, as Wright and Bernard supposed, essential to the function of this fluid. Acids, whether organic or inorganic, did not interfere with its action.

Jacubowitsch mixed a dog's pure filtered gastric juice, free from histological elements, neutralized by strongly alkaline filtered human saliva, with a fresh decoction of starch. He acidified another portion of the saliva with the same gastric juice. Both mixtures, after standing two hours at a temperature of 100° F., gave decided indications of sugar with Trommer's test.

He also took pure filtered gastric juice, and (1) mixed it with saliva to neutralization; (2,) made it alkaline by excess of saliva; (3,) made it alkaline by soda; (4,) acidified saliva with it; (5,) mixed it with starch. All these mixtures, except 3 and 5, when kept at a temperature of from 86° to 104°, showed distinct traces of sugar in fifteen minutes. Gastric juice, therefore, while it is powerless of itself, does not deprive saliva of its saccharifying power. Bidder and Schmidt fully corroborate these statements.

In a question, however, which seems, at first sight, identical, they differ from Jacubowitsch. They failed to detect any sugar in the stomach, after starch had been introduced by an œsophagus tube or through a gastric fistula. Yet, when the gastric fluid is alkaline, in consequence of the quantity of saliva which the animal has swallowed, or when it exhibits large particles of frothy saliva, it occasions the immediate metamorphosis of the starch ; while, when the gastric juice is acid, and little saliva is present, the change does not take place in less than an hour-and-a-half, and then but slightly.

There are but two ways of explaining this discrepancy. One is, that the sugar has been immediately converted into lactic acid, and so lost in the contents of the stomach. The other is, that it has been absorbed as fast as formed. Difficulties attend both explanations, but the latter is the more probable.

It must be stated, however, that other chemists do not agree with Bidder and Schmidt. Frerichs, "in at least fifty experiments," constantly found sugar in the stomachs of animals. Jacubowitsch fed a dog, suffering with gastric fistula, on starch, after twelve hours fasting. Four or five hours afterwards, sugar was always present. In another dog, whose salivary ducts had been tied, but in which all the other circumstances of the experiment were identical, no conversion of the starch into sugar, or even into dextrine took place.

Lehmann always found sugar in the stomachs of animals fed on starch.

In the other carbohydrates, saliva exerts no definite metamorphic influence. Cane sugar, gum, cellulose and bassorin, remain unchanged in the saliva ; in certain species of sugar only, after long continued digestion, at a high temperature, formation first of lactic and then of butyric acid takes place.

In reference to the *sulphocyanide of potassium*, Kletzinsky found, that it retarded fermentation generally, and killed the low fungoid growths which result from it. In certain children, whose mouths contained fungous formations, the saliva was totally deficient in *sulphocyanides*.

---

18.—*Gastric Secretions.*—"Observers are pretty generally agreed that there are two distinct secretions in the stomach, the *gastric*

*juice* and the *gastric mucus.* The former is a clear, watery, or slightly viscid fluid, containing only a few accidental morphological elements, such as gastric cells and their nuclei, and a granular substance, resulting from the disintegration of these elements. It is only secreted during digestion, and it alone, of all the gastric fluids, possesses any digestive power. The latter accumulates in the stomach during the intervals of digestion. It is neutral in its reaction, and exerts no solvent power upon the protein-compounds.

Frerichs, however, states that, at the commencement of digestion, the round cells in the interior of the glands escape in excessive quantities, and form a stratum of about a line in thickness, which has hitherto been regarded as mucus, and which either invests the interior of the stomach or surrounds the contents in the form of a white membrane, the latter being especially the case when dry food is taken. The gastric cells become gradually disintegrated during the continuance of the digestive process, and thus afford a continuous source of pepsin or ferment. The digestive act being accomplished, the gastric glands collapse, and in that state no nuclei or cells, and only a few scattered granules, escape from them. During abstinence from food, the morphological elements are again perfectly formed, and the tubes become filled with cells, which, probably, in very prolonged fasting, again become disintegrated.

Kölliker has called attention to two facts connected with digestion. One is, that during this process, in many of the mammalia, *the gastric mucous membrane is covered with a more or less thick coat of mucus.* He has found this mucus constantly present in certain animals, while, in others, it is altogether absent, or present only in very small quantity. When present, it does not consist solely of gastric cells, and often, indeed, contains no trace of them. In the pig and rabbit it consisted chiefly of desquamated cylindrical epithelium.

The second fact is, that in many animals *the gastric glands occur in two different forms, and yield two different kinds of secretion.* One set of these glands is lined by cylindrical epithelium, the other set contains roundish cells. Goll found that the digestive powers of the fluids secreted by these two classes of glands, were widely different. Acidified mixtures, prepared from the glands with round cells, very rapidly dissolved coagulated protein-compounds, while those prepared from the glands with cylindrical epithelium, either exerted no action whatever, or only a very slight action, after a long period.

Köllicker's views are stated by Dr. Day as follows: "A very acid reaction is invariably observed in that part of the gastric mucous membrane in which the complicated glands lie, and the so-called pepsin must necessarily be situated here, since the mucous membrane of this part yields an energetic digestive fluid; indeed, it could hardly be presumed that pepsin was secreted by the glands lined with cylindrical epithelium, and the very slight effects which were occasionally observed to be slowly produced by the digestive fluid prepared from these glandular structures, were probably due to the presence of the nitrogenous matters which occur here, for all mucous membranes yield, with distilled water and a little acid, a fluid possessing *some* solvent power. The acid reaction exists in the whole stomach, and the more simple glands, which do not secrete pepsin, may possibly yield an acid juice; but the reaction is always weakest where merely the simple glands are situated. The glands with cylindrical epithelium give forth a secretion containing no visible objects, excepting a few epithelium cells from their upper part, while the true gastric glands, at all events, in certain animals, yield not only cylindrical, but also roundish cells. These latter cells, however, do not seem to be invariably present in gastric juice, and in many animals this secretion apparently contains no visible particles. There can be no question that it is these comparatively large, round cells, which secrete the pepsin, which either oozes through their walls, or is liberated by their solution.

19.—*Chemical Characters of Gastric Juice.*—Fresh gastric juice, even when the animals have been kept for twenty-four hours, or longer, without food, is never perfectly pure, but always contains hair, sand, remnants of food, &c. It often escapes from the canula in large drops, as a perfectly clear and limpid secretion, but the impurities soon reappear, and give color to the fluid. This color varies with that of the ingesta. In the dog, it is ash-grey; in the sheep, green or olive. The filtered fluid is always clear and transparent. The substances left on the filter, when examined microscopically, were found to consist of partially digested muscular fibre, fat, vegetable cells, &c. Epithelial cells are rarely found, but round corpuscles are there in abundance. These elements are merely accidental, as the gastric juice, when filtered, exerts an equally energetic solvent action upon the substances which are presented to it.

To determine the acidity of the gastric juice, these observers treated it with a normal solution of potash, containing 1 per cent. of potash. From nine experiments on a dog, after ligature of the salivary ducts, it appeared, that on an average, 100 parts of the juice were neutralized by 0.356 of potash; while in eleven, whose salivary ducts had not been tied, the average amount of potash required for neutralization, was 0.390 of potash to 100 of juice. This paradox is explained by the circumstances of the diet of the two sets of dogs, the first having been fed on albuminous, the latter on amylaceous food. There is, however, always very great variation in the acidity of this secretion.

The question of the particular acid on which this reaction depends, has also been taken up by these physiologists. It was originally, as is well known, regarded as hydrochloric acid. Lehmann, however, advanced the opinion, that lactic acid was the acidifying principle, and this was confirmed by quite a number of eminent physiological chemists. Hübbenet, however, Bidder and Schmidt and Graham, favor the old view. Schmidt not only discovered hydrochloric acid, but demonstrated most positively the absence of lactic acid in the cases on which he experimented. Lehmann, on re-examining the matter, repeats his former opinion, and distinctly shows the presence of lactic acid. So the matter stands at present. On both sides of the question are ranged men of the greatest distinction, and there can be no possible doubt that, in the distinct examinations they have made, their reports are correct. The only way of accounting for the discrepancy is, to suppose that both these acids are secreted by the gastric mucous membrane, though the causes which determine the separation of one in preference to the other, on any particular occasion, remain unknown.

*Pepsin,* the organic substance which exerts so powerful an influence over digestion, has been very carefully studied. It is precipitated by bichloride of mercury and acetate of lead, and is decomposed by anhydrous alcohol and a boiling heat. It may be separated from its mercurial and plumbeous compounds by sulphuretted hydrogen, and when obtained, it communicates to hydrochloric acid a powerful solvent influence upon albuminous matters.

Schmidt supposed that it formed, with hydrochloric acid, a conjugated acid, and still adheres to that view. He calls the active portion of the gastric juice *pepsin-hydrochloric* acid, and says, that it resembles ligno-sulphuric more than any other acid. Lehmann, however, gives good reasons for considering this position untenable.

The ultimate composition of pepsin, according to Schmidt, is, carbon 53.0, hydrogen 6.7, nitrogen 17.8 and oxygen 22.5.

The mean composition of gastric juice will be found in the following table. A is a mean of 9 experiments on the gastric juice of a dog, whose salivary ducts had been tied. B is a mean of 3 analyses of this fluid in a dog, whose salivary ducts had not been tied, and C is taken from two analyses of a sheep's gastric juice:

| | Gastric Juice, (without saliva.) A. | Gastric Juice, (with saliva.) B. | Gastric Juice, sheep. C. |
|---|---|---|---|
| Water, | 937.062 | 971.171 | 986.147 |
| Solid residue, | 26.938 | 28.829 | 13.853 |
| | 1000.000 | 1000.000 | 1000.000 |
| Ferment or pepsin, | 17.127 | 17.336 | 4.055 |
| Hydrochloric acid, | 3.050 | 2.377 | 1.234 |
| Chloride of potassium, | 1.125 | 1.073 | 1.518 |
| Chloride of sodium, | 2.507 | 3.147 | 4.369 |
| " " ammonium, | 0.468 | 0.537 | 0.473 |
| " " calcium, | 0.624 | 1.661 | 0.114 |
| Phosphate of lime, | 1.729 | 2.294 | 1.182 |
| " " magnesia, | 0.226 | 0.323 | 0.577 |
| " " iron, | 0.082 | 0.121 | 0.331 |

20.—*Action of Gastric Juice.*—Bidder and Schmidt have examined this subject under six distinct inquiries:

1. The part taken by the saliva in the gastric digestion of nitrogenous bodies.

2. The influence of a greater or less quantity of free acid in the gastric juice.

3. The action of neutralized or alkaline gastric juice.

4. The difference of the action of the filtered and unfiltered gastric juice, with the view of ascertaining the influence of the organic histological elements suspended in it.

5. The influence of greater or less dilution and concentration of the gastric juice on its solvent power.

6. The influence of heat, addition of bile, &c., on the digestive power of the fluids of the stomach.

The following results were obtained:

1. Saliva does not, as Wright supposed, assist in the digestion of albuminous matters. Indeed, more dry albumen was digested by dogs after ligature of the salivary ducts than before. The average of 27 experiments showed that the matters digested, when the salivary glands were tied, in two, four and nine hours, were 29, 62 and 76 per cent.; and when they were not tied, the averages of the same number of experiments for equal times, were only 26, 45 and 65 per cent. This difference, probably, depends upon the neutralization of the gastric juice by the alkaline saliva.

2. The acid gastric juice was shown to be the agent in digestion, and the difference between the times required for full solution in the stomach and out of it, depends upon the motions of the organ, bringing the food constantly in contact with fresh portions of gastric juice. Several of these experiments show that, provided a sufficient quantity be present, the digestive power of the gastric juice is directly proportioned to the quantity of free acid present. The neutral or alkaline gastric mucus of fasting animals, dissolved only very small quantities of albumen, but on adding hydrochloric acid, the activity of the fluid was at once augmented.

3. When the free acid is neutralized by potash, the digestive powers of the gastric juice are entirely destroyed.

4. The influence of filtration was by no means marked. They found that gastric juice, deprived of all its solid organic matters, was just as efficacious as that which had not been filtered. Frerich's view, however, differs from this. He thinks that new ferment is constantly developed by the action of the free acid from the cells contained in the gastric juice.

5. No definite results were obtained.

6. Rapid ebullition or evaporation at a boiling heat destroyed the solvent power of gastric juice. When, however, this fluid was frozen, its digestive power remained. Bile mixed with gastric juice completely destroyed its solvent power upon albumen, even while the fluid remained acid.

---

21.—*Quantity of Gastric Juice.*—Bidder and Schmidt found that dogs secrete, in twenty-four hours, an amount of gastric juice equal to one-tenth the weight of the body. If the same relation exists in man between the entire weight and the amount of this fluid, a man of ordinary weight would secrete from 14 to 17 pounds. Lehmann's estimate differs from this. The following are his data:

100 grammes of the fresh gastric juice of a dog cannot, (according to his experiments,) dissolve more than 5 grammes of coagulated albumen, calculated as dry.

An adult man receives into the stomach about 100 grammes of dry albuminous matter in twenty-four hours.

Hence, to digest this quantity, there must be secreted 2000 grammes, or 4 pounds of gastric juice.

Bidder and Schmidt's estimate of the amount of solid albumen which 100 grammes of gastric juice will dissolve, is much lower than Lehmann's. Their maximum is but 3.95, less than Lehmnan's minimum, which is 4.317. His maximum is 6.14. Their average, (a mean of twenty-seven experiments,) is 2.2.

They attach great importance to the extraordinary amount of this very aqueous secretion. It so dilutes the dissolved nutriment "that it is enabled to enter into endosmotic relations with the blood circulating in the intestinal walls, and even with the chyle in the lacteals. Hence, within certain limits, the quantity of secreted gastric juice must be greater, the less fluid is directly introduced into the stomach as drink."

22.—*Bile.*—Their first inquiry was, whether the bile was an excretion or not?

This they have not positively determined. They found, in several instances, that the animal gradually lost health and strength, and finally died, after the passage of bile into the intestine had been prevented. Where the spleen was tied, the decline of health and strength was more rapid. In one case, however, in which the spleen was not tied, an emaciated dog, with a biliary fistula, recovered his health and strength. He ate, however, more food than was natural to him in an unmutilated state. The ductus communis choledochus subsequently became re-established, and then he rapidly increased in weight. In those animals which died, there was a manifest tendency of the contents of the bowels to putrefaction, but the most marked change was the gradual disappearance of the fat, the muscles remaining in their ordinary condition.

23.—*Quantity of Biliary Secretion.*—The amount of bile secreted varies greatly in different animals. The following table exhibits this, by showing the amount for each kilogramme of weight, in different animals:

| | Cats. | Dogs. | Sheep. | Rabbits. | Geese. | Rooks. |
|---|---|---|---|---|---|---|
| Fresh bile, | 14.500 | 19.990 | 25.416 | 136.84 | 11.784 | 72.096 |
| Dried residue, | 0.816 | 0.988 | 1.344 | 2.47 | 0.816 | 5.256 |

Of course no inference can be drawn from these experiments as to the quantity secreted by man. Bidder and Schmidt think it probable that an adult man secretes daily a kilogramme and a half, about 54 ounces,) containing 5 per cent. of solid constituents.

They found that the secretion was increased during digestion. It reached its maximum 13 or 14 hours after eating. In prolonged starvation it gradually and progressively diminishes.

Nasse showed that the *nature of the food* exerts an influence over this secretion. A flesh-diet induces a far more abundant secretion than amylaceous food. Thus, a dog fed on bread and potatoes, secreted daily 171.8 grammes of bile, containing 62.52 grammes of solid matters; but, when kept on a flesh-diet, secreted in the same period, 208.5 grammes of bile, containing 70.6 grammes of solid matters. Bidder and Schmidt found that in cats, fed on fatty matters exclusively, the secretion of bile was as much diminished as though they had fasted the same length of time.

Nasse found that large doses of carbonate of soda very much diminished the secretion of bile, especially of its solid constituents, while calomel augmented the quantity of fluid bile, but caused a diminution of its solid constituents. All of these observers found that, during fever, the amount of this secretion was always diminished.

Pure bile is always clear, epithelium being only found in it during catarrh. Pure yellow bile may be rendered green by oxygen, and green bile may be made yellow by deoxydation. In the gall-bladder, the bile is changed in color and gets purer in water and fuller of mucus. Thus, when the fresh bile of animals contained, on an average, 5 per cent. of solid constituents, that in the gall-bladder contained as much as 10 or 20 per cent.

The reaction of pure bile is always neutral. When mixed with the mucus of the gall-bladder, it becomes alkaline, and when decomposed, it sometimes develops free acid.

The question of the *resorption* of bile was also examined. A dog was fed on black bread, then on flesh, then on black bread again. So different were the fæces produced by these two sorts of diet, that the kind of food could be easily determined by an inspection of the dejections. During the flesh-diet, but little bile could be detected in the fæces. This proximate analysis was confirmed by a determination of the elements, little sulphur being found in these stools. These observers are, therefore, led to conclude, contrary to the opinions of Mulder and Frerichs, that by far the greatest portion of the solid biliary matter, probably seven-eighths, is returned into the mass of the juices.

The *antiseptic* properties of bile were shown by the great putridity of nitrogenous, and the excessive acid fermentation of amylaceous food. Hünefeld's statement, that bile free from mucus digests blood-corpuscles, but that normal bile possesses no such power, is confirmed by these observers. They also show that bile exerts no influence over the digestion of albuminous or amylaceous ingesta.

In regard to its influence on the *resorption of fat*, they found that though fat might be taken up when there is no bile in the intestine, yet the presence of that secretion greatly facilitates this action. The chyle also is found to be relatively poor in fat, after ligature of the ductus communis choledochus. Bile seems to render the intestinal membrane more permeable to fat. Oil rises higher in a capillary tube, moistened with bile, than in a similar tube, not wet with this secretion.

These experiments appear to show that bile is not, at any rate, a very active digestive fluid. The length of time after food, at which this secretion reaches its maximum, is too great for it to exert much influence over chylification.

Bidder and Schmidt think that its principal object is "to prolong the series of changes to which animal matter is subjected in the organism, and thus to render it for a longer time efficient in the discharge of vital processes," in short, to economize animal tissue.

24.—*Pancreatic Juice.*—This fluid is a clear, limpid, colorless, transparent, viscid and ropy fluid, containing no microscopical particles. It has a strong alkaline reaction, and on the addition of 85 per cent. alcohol, is coagulated into a milky mass, which deposits thick white flakes, above which is a clear, colorless, strongly alkaline alcoholic solution. The flakes, when collected by filtration, washed with alcohol and dried, may be, for the most part, redissolved in water, and then they constitute a solution resembling the original secretion. Strongly heated, they leave a small residue of carbonate of lime. The alcoholic solution, when evaporated, leaves a colorless albuminate.

According to Schmidt, its specific gravity is 1.031, while, according to Lehmann, it ranges from 1.008 to 1.009. The following are the analyses of the fluid:

Pancreatic Juice of the Ass, (Frerich's.)

| | | |
|---|---|---|
| Water, . . . . . . | | 986.40 |
| Solid residue, | | |
| Fat, . . . . . . | 0.26 | |
| Alcohol extract, . . . | 0.15 | |
| Water extract, . . . . | 3.09 | |
| Soluble salts, . . . . . | 8.90 | |
| Insoluble salts, . . . . | 1.20 | |
| | | 13.60 |
| | | 1000.00 |

Pancreatic Juice of Dog, (Schmidt.)

| | | |
|---|---|---|
| Water, . . . . . | | 900.76 |
| Solid residue, | | |
| Organic matter, . . . . | 90.38 | |
| Inorganic constituents, . . | 8.86 | |
| | | 99.24 |
| | | 1000.00 |

In another case, Schmidt found that the solid matters in 1000 parts amounted to 115.6.

The water extract of Frerichs, or the organic matter of Schmidt, resembles albumen and casein in many of its properties, but is not identical with albuminate of soda, casein or ptyalin.

The *quantity* secreted is unknown. Bidder and Schmidt think, that for every kilogramme of weight, a dog secretes 0.10 of a gramme, and over. According to this, a man weighing 64 kilogrammes (about 140 pounds,) would secrete, in twenty-four hours, 150 grammes of pancreatic juice, yielding 15 grammes of solid residue.

Weinmann, who has been also experimenting on this subject, obtains results which differ from these. He found that a dog secreted 35.184 grammes to every kilogramme of weight.* The secretion is at its minimum after long fasting, and at its maximum

*According to this, a common sized man would secrete 11½ pounds in twenty-four hours.

after food, especially after water. The proportion of solids does not appear to vary so widely as the secretion itself.

During fasting, the gland is pale and anæmic, but during digestion, it becomes highly vascular and turgescent, and yields a copious secretion.

They prove that the fluid exerts no influence over the digestion of albuminous substances, but has a powerful saccharifying action on starch. They consider it more energetic in this respect than saliva. The substance upon which this action depends, exists ready formed, in the pancreatic juice, and retains its energy even after that fluid has stood for twenty-four hours at a temperature of 64°. The action is not arrested by gastric juice or bile.

The *digestive power* of this fluid on fats has been variously stated. That the fatty acids are not liberated from their bases, as the Germans generally have understood Bernard to declare, no one can now doubt. This change, however, takes place readily out of the body. Lenz has shown that the acid gastric juice is the impediment to this metamorphic action within the body.

It must be confessed that much doubt still overhangs this whole subject. But little light is cast upon it by the pathological fact of the occasional discharge of fatty matters from the intestines. This was formerly thought, by Dr. Bright, to depend upon pancreatic disease. Since Bernard's discoveries, this opinion of Dr. Bright's has gained ground among physicians, but it so happens that the distinguished author of it has abandoned it as a too hasty inference. Various lesions have been found to accompany this symptom, so that pathology leaves us just where physiology does, still very much in the dark.

25.—*Intestinal Juice.*—Much diversity of opinion, in regard to this matter, exists among authors. All that can be certainly affirmed with regard to it, is that, after filtration, it is colorless, tenacious and ropy, with a strong alkaline reaction.

Frerichs found that it effected no change in protein-compounds or gelatigenous substances, disintegrated fat only, as all viscid fluids do, and exerted no special influence over starch. Hence, he does not regard this substance as a special digestive agent.

Lehmann, however, found that it possessed, in a high degree, the power of converting starch into sugar, but that fats and protein bodies were scarcely, if at all, affected by it.

Zander and Bidder and Schmidt, on the other hand, find, as the result of experiments on nineteen cats and two dogs, that the intestinal juice digests albuminous bodies almost as well as the gastric secretion, and that its saccharifying power over starch nearly equals that of saliva or pancreatic juice.

---

### MISCELLANEOUS ORGANIC CHEMISTRY.

26.—*Chloroform.*—Professor Horsford, of Cambridge, read, at the last meeting of the American Association for the advancement of Science, an article on the fatal effects of chloroform.

It will be remembered, that Dr. Charles T. Jackson, some time since, made a great outcry about fusel oil, to which he attributed the deaths occurring after the administration of chloroform. This oil is always present in badly rectified whiskey, and is also to be found in poor specimens of alcohol. It was supposed that the deadly chloroforms were made from this whiskey or the impure alcohol, that the fusel oil formed with the bleaching salt a volatile compound which passed over, contaminated the chloroform and communicated to it poisonous properties. This subject has been examined by Prof. Horsford, and he has communicated his results in the article before us. Another hypothesis was, that chloroform was susceptible of spontaneous change, which unfitted it for use as an anæsthetic.

Chloroform was sold, some of which was impossible to be inhaled, by reason of the presence of free chlorine and hydrochloric acid, and other samples yielded an offensive and putrid odor.

The bad chloroform examined by Prof. Horsford, was yellowish-green, as was the air above it in the bottle. Floating on its surface was a thin layer of deep yellow, oily matter. The whole smelt strongly of chlorine.

A quantity of this liquid, inverted in a test-glass over mercury, evolved gases, at first greenish and afterwards colorless, which gradually forced down the chloroform, till, after nine months, several cubic inches of gas were formed from a cubic inch of the liquid. It was easily purified by soda-lime, and by placing it, for a few days, in contact with candle-wick.

The oil was examined for sulphur and nitrogen, and gave a negative result.

Chloroform was then manufactured with alcohol containing fusel oil, in varying proportions, but in every case the resulting chloroform was good. Pure fusel oil was then treated with water and bleaching salt, in the same manner as alcohol in the manufacture of chloroform, and a product was obtained resembling fusel oil in smell, but having a different specific gravity. This varied from 0.8224 to 0.8236, while that of pure fusel oil is 0.8124. It contained 1.35 per cent. of chlorine, which was due to the chloroform derived from the trace of alcohol present in the fusel oil. Alcohol containing impure methyl alcohol (wood spirit) gave good chloroform.

Experiments were made with the distillate obtained from fusel oil, water, and bleaching salt, to determine its effects upon the economy. The result was, that after a protracted application of the agent, a very slight lethargic state was induced, from which, however, the animals speedily and perfectly recovered.

Above fifty different preparations of chloroform were made, from which, together with the above experiments, the Professor deduces the following conclusions:

"1st. That good chloroform does not spontaneously change in a period of nine months.

"2d. That the bad chloroform, containing free chlorine and hydrochloric acid, may be produced by using a bleaching salt of great strength, with a quantity of alcohol disproportionately small.

"3d. That the bad chloroform may be produced by receiving the distillate into water, so as immediately to withdraw the alcohol from the chloroform.

"4th. That bad chloroform may be produced by passing chlorine directly into chloroform.

"5th. That no formula for its manufacture can be relied upon as a guide, since bleaching salts vary in strength when derived from different factories, and vary with age. In the foregoing experiments, the range is 15 to 30 per cent.

"6th. That quick lime added to the mixture does not promote the economy of manufacture.

"7th. That the chlorine and hydrochloric acid of bad chloroform, as observed by Dr. Dwight, may be removed by agitation with a little alcohol.

"8th. That the ill effects observed in the administration of chloroform, are not due to the presence of chlorine, as the irritation is such, when it is attempted to inhale it, as to prevent inhalation altogether.

"9th. That the ill effects are not due to any poisonous product arising from the action of bleaching salt on the small quantity of fusel oil, in the alcohol employed in the manufacture of chloroform.

"10th. That the ill effects are due to peculiarities of constitution or temperament of some patients, and in a few rare cases, to want of attention or judgment on the part of the person administering it."

[The result of the whole is, that we know nothing positive, as yet, of the counter-indications to the administration of this anæsthetic. There is danger in its use, and we cannot, as yet, determine where that danger lurks, or on what it depends.]

12.—*Fusel Oil.*—The Journ alof the Franklin Institute, for June, publishes a memoir by Dr. Charles H. Wetherill upon this oil, which has acquired new interest since Dr. Jackson, of Boston, has attempted to account for the occasional poisonous properties of chloroform by its supposed presence in the noxious drug.

This oil, as is well known, was first obtained by Scheele from fermented potatoes, and examined carefully by Dumas, Pelletan and Cahours, to the last of whom we are principally indebted for what we know about it. It always contains amylic alcohol and has been described as synonymous with that substance.

The common oil, however, contains a variety of other substances. Mulder detected in it ænanthic, Kolbe, margaric, and Rowney, capric acid. Kent found in it acetic and valerianic acids and an ether, the acid of which was not isolated.

Wurtz isolated from it butylic alcohol.

Wetherill detected acetic and caprylic acids, and oil of turpentine, which was probably an accidental contamination. He obtained indications also of the presence of formic, caproic and ænanthic acids.

13.—*Organic Poisons.*—M. C. Flandin has published a memoir in the *Comptes Rendus* in which he proposes a new method of testing for the organic poisons.

He mixes 12 parts of anhydrous lime or baryta with 100 of the substance to be examined, pounds them together in a mortar, heats the mixture to 212°, pulverizes again, and treats the powder three times with boiling anhydrous alcohol. He filters the liquid, which passes through colorless, and contains nothing but the proximate principle, mixed with fatty or resinous matters. The alcohol is now slowly evaporated, and the dry residue treated with ether to remove the fats. If the principle be insoluble in ether, it is easily obtained by filtration or decantation. If soluble in that menstruum, it must be dissolved by some special substance, as acetic acid, and precipitated with ammonia.

M. Flandin has made several experiments to test the accuracy of this process, and has succeeded in obtaining a cognizable amount of these poisons from admixtures of minute quantities of them with animal matters. In one instance he mixed 10 centigrammes of morphia with 100 grammes of flesh, and obtained several centigrammes of morphia from the mass after several months putrefaction.

14.—*Tobacco.*—The Lancet is publishing a series of papers on the weed, which contains very full information upon some subjects connected with its growth, manufacture, chemical composition and adulterations.

Vauquelin found in it, nicotina; albumen; red matter soluble in alcohol and water; acetic acid; supermalate of lime; chlorophyll; nitrate of potash and chloride of potassium; sal ammoniac, and water. The leaves contained also woody fibre, oxalate and phosphate of lime, oxyd of iron and silica.

Conwell found in it gum; mucilage soluble in both water and alcohol; tannin; gallic acid; chlorophyll; green pulverulent matter, soluble in boiling water; yellow oil, having the odor, taste and poisonous properties of tobacco; pale yellow resin in large quantity; nicotina; a substance analogous to morphia; an orange red coloring matter; nicotianin.

Posselt and Reinman have made a quantitative analysis of the fresh leaves, which we copy:

| | |
|---|---|
| Nicotina, | 0.060 |
| Concrete volatile oil, | 0.010 |
| Bitter extractive, | 2.870 |
| Gum with malate of lime, | 1.740 |
| Chlorophyll, | 0.267 |
| Albumen and gluten, | 1.308 |
| Malic acid, | 0.510 |
| Lignin and a trace of starch, | 4.969 |

| | |
|---|---|
| Salts, (sulphate, nitrate and malate of potash, chloride of potassium, phosphate and malate of lime, and malate of ammonia,) . . . . . . | 0.734 |
| Silica, . . . . . . . . . . . | 0.088 |
| Water, . . . . . . . . . . . | 88.280 |
| | 100.836 |

The most important of these constituents are *nicotina* and *nicotianin.*

Nicotina is found not only in the leaves, but in the roots, seeds and smoke of tobacco. It is obtained by digesting an aqueous extract of the leaves in rectified spirit, which takes up the nicotine in combination. The tincture is concentrated and decomposed with potash. This sets free the nicotine which may be taken up again with ether. To purify it, oxalic acid is added to the ethereal solution; the precipitate of oxalate of nicotine which falls, is repeatedly agitated with ether, and again decomposed by potash and ether. The ethereal solution is now distilled in a salt-water bath, transferred to a retort, through which a current of dry hydrogen gas is made to circulate, heated in an oil bath, first to 284° F., to get rid of water, ether and ammonia, and then to 356° F., to distil over the nicotine. This process easily gives 4 per cent. from Virginia tobacco.

It is a colorless liquid, with an acrid odor and an acrid, burning taste. It boils, and is decomposed at 482° F., becomes brown on exposure to the air, and readily burns with the aid of a wick. It is soluble in water, ether, alcohol and the oils. With the acids, it forms salts which are usually very acrid, crystallizable, and have the taste and smell of tobacco. Chloride of mercury (corrosive sublimate) throws down a white flocculent precipitate, and bichloride of platinum a yellow granular one, from solutions of nicotina.

*Nicotianin,* the concrete volatile oil of tobacco, is obtained by distillation. Six pounds of the leaves yield eleven grains of oil. It has the odor of tobacco and a bitter taste. It produces in the throat a sensation similar to that excited by tobacco smoke. Applied to the nostrils, it causes sneezing. Helmstädt swallowed a grain of it, which produced nausea, giddiness, and an inclination to vomit.

*Tobacco smoke* has been analyzed by Raab, Unverdorben, Zeise and Melseur.

Raab found in it, carbonate of ammonia; acetate of ammonia; nicotianin; empyreumatic oil; carbonaceous matter; moisture and several gases.

Unverdorben obtained, by dry distillation, a volatile oil; an oleaginous acid; an empyreumatic acid; resin; traces of a powder insoluble in potash and acids; odorin, a small quantity; a base soluble in water, (nicotine ?); fusein; red matter soluble in acids; and two extractive matters, one forming a soluble, the other an insoluble compound with lime.

Zeise detected a peculiar empyreumatic oil; butyric acid; carbonic acid; ammonia; paraffine; empyreumatic resin; water; acetic acid; carbonic oxyd; and carburetted hydrogen.

Melsens found nicotina in tobacco smoke.

It is evident from these analyses, that the notion of tobacco preserving the teeth, at any rate when smoked, is an extremely erroneous one. The smoker brings in contact with his teeth, at a high temperature, acids which cannot fail to attack the phosphate of lime which forms their principal mineral basis.

---

15.—*Nicotin.*—We find in the *St. Louis Medical and Surgical Journal*, for March, a translation from the *Prager Vierteljahresschrift*, which contains some important additional information in reference to this agent, chiefly in regard to its physiological action.

Albers, of Bonn, has instituted a series of experiments to compare this poison with hydrocyanic acid. The latter stupifies in 30-45 seconds, and does not produce its final action till after 30-60 seconds. Nicotin manifests its action in 10-15 seconds, and completes it in 15-35 seconds. It is therefore much more rapid in its operation than hydrocyanic acid. This seems to have some bearing on the theory of nervous transmission of therapeutical or toxic impressions. According to Black's experiments, the shortest time in which a remedy can enter the blood, is three-quarters to one-and-a-quarter minutes.

Secondly, The action of nicotin, short as it is, is always very painful. "As soon as the poison touched the tongue, the rabbit began to cry and even more continually, than Albers has ever heard it in dissecting a nerve; it is known, however, that these animals do not cry but in the highest degree of pain. There was found on that part of the tongue, upon which the drop of nicotin had fallen, a yellow spot, and the smell of nicotin.

Thirdly, The nicotin acts upon the entire motor apparatus, the superior as well as the inferior. The difficult respiration, attendant on poisoning by prussic acid, was absent in cases of death from nicotin. Nicotin paralyzes the motions, but does not affect the sphincters, the bladder having been found filled with urine, after death by it. In frogs, the action of nicotin differs still further from that of prussic acid, which paralyzes the anterior extremities first. This recalls Flourens' assertion that the motor power over the anterior extremities resides in a portion of the nervous axis different from that which presides over the movements of the posterior limbs.

Fourthly, However speedily these agents kill, the animal irritability lasts long after the heat has ceased to beat. The muscles have contracted twenty-five minutes after the cessation of respiration.

Vandercorput, of Brussels, has instituted experiments with nicotin, which had been kept two years. The smallest quantity of it, put into the mouth caused a burning sensation resembling a severe laceration, and communicated the taste of a cigar moistened with saliva. In the pure alkaloid the smell is feeble, but is easily developed by the addition of caustic ammonia. Four drops of nicotin put in the mouth of a large dog killed him in less than a minute. He made a few leaps, and fell on the left side. Acetic acid was immediately applied, but failed to restore him. A drop in the eye of a pigeon killed the bird almost instantaneously. The legs were spasmodically extended and it fell on the left side. The corner of the poisoned eye was slightly opaque and its pupil normal, while that of the other eye was dilated.

According to Albers, birds die most rapidly, while a frog after receiving a drop of nicotin lived more than two minutes. Another frog, which got a dose of nicotin mixed with acetic acid, did not appear at all affected; the application of a little caustic potash, however, immediately caused convulsions of the posterior extremities and death followed instantaneously.

Vleminckx, of Brussels, has experimented upon two guinea-pigs, a cock, two dogs and a cat, and the following are his conclusions:

1st. The poisoned animals fall sometimes on the right, sometimes on the left side.

2d. The poison is more active applied to the eye than to the mouth.

3d. The most constant symptoms are congestion of the *pia mater* and the lungs.

Albers could not confirm the statement of congestion of the lungs.

